# Tyrosine Kinase Inhibitors for Non-Small Cell Lung Cancer and Eye Metastasis: Disease Relapse or a New Entity?

**Published:** 2016

**Authors:** Paul ZAROGOULIDIS, Sofia LAMPAKI, Panos CHINELIS, George LAZARIDIS, Sofia BAKA, Aggeliki RAPTI

**Affiliations:** 1 Pulmonary Department, Oncology Unit, G. Papanikolaou General Hospital, Aristotle University of Thessaloniki, Thessaloniki, Greece; 2 Oncology Department, G. Papageorgiou University General Hospital, Thessaloniki, Greece; 3 Oncology Department, Interbalkan European Medical Center, Thessaloniki, Greece; 4 Pulmonary Department, Sotiria Hospital of Chest Diseases, Athens, Greece

**Keywords:** Tyrosine Kinase Inhibitor, Non-Small Cell Lung Cancer, Eye Metastasis

## Abstract

Lung cancer is still diagnosed during the advanced stage of the disease and most patients do not have the opportunity for surgical treatment, despite the new diagnostic equipment that has been made available in recent years, such as the radial and linear endobronchial ultrasound (EBUS) and electromagnetic fiberoptic bronchoscopy. However, novel targeted therapies with second generation tyrosine kinase inhibitors and immunotherapy are available. In this commentary, we will focus on eye metastasis after initiation of tyrosine kinase inhibitors due to epidermal growth factor mutation of lung cancer adenocarcinoma.

## INTRODUCTION

**Figure 1:**
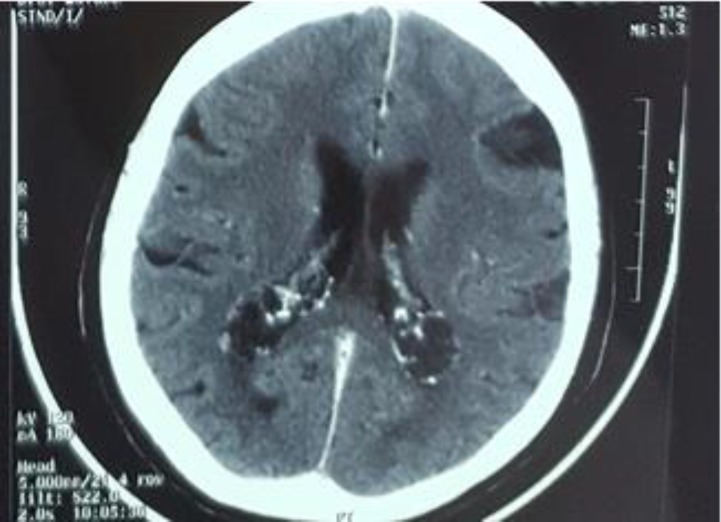
CT scan of the brain after irradiation

Lung cancer still remains the leading cause of cancer in male cancer patients and very soon it will be the leading cause of cancer death among women (1). Disease symptoms are such that patients are not diagnosed until they are in an advanced stage. Since most patients are smokers, a non-specific cough is usually attributed to their smoking habit. Only patients with hemoptysis are typically worried and seek medical attention. We still do not have a blood test that could be used as an early diagnostic marker, such as those used in prostate or gastrointestinal cancers (2). Non-small cell lung cancer treatment had a breakthrough in the past 15 years with tyrosine kinase inhibitors, specifically with the first generation erlotinib and gefitinib and, currently, with the second generation afatinib. These agents are referred to as ``targeted`` therapy since they target epidermal growth factor mutations in lung cancer adenocarcinoma patients (3-8). The most common side effects of these agents are skin rash and gastrointestinal disorders, making them safer as therapy. It has been observed that more severe side effects are associated with a higher treatment efficiency. In the past two years, clinical physicians have also searched for epidermal growth factor mutations in mixed non-small cell lung cancer patients (adenocarcinoma and squamous, or squamous alone) in an effort to determine whether tyrosine kinase inhibitors would be effective in these patients (9, 10). Current guidelines indicate that for epidermal growth factor mutations, these agents should be used as first-line treatment. However, disease relapse has been observed by many clinical physicians during the course of treatment. ``Relapse`` still has not been correctly identified in patients receiving chemotherapy. For example, we are still evaluating these patients with the response evaluation criteria in solid tumors (RECIST), even though we are not sure if these criteria should be applied to these patients (11-13). A very serious issue that has not been answered is whether all tumor sites have epidermal growth factor receptor (EGFR) mutations. In tissue samples from the primary site, we typically find that the newly diagnosed adenocarcinoma is EGFR positive; however, we do not know if the metastatic sites are also EGFR positive. We assumed that they are and we studied the response using the RECIST criteria. There are some treatment proposals for the treatment options after disease relapse when a patient receives tyrosine kinase inhibitors (TKIs). In regards to targeted therapy options, there is the option of a re-biopsy of the primary lesion or at a new metastatic site. If the T790 mutation is observed, then osimertinib is a treatment option (14). It has also been reported that crizotinib, another TKI that is used to treat an anaplastic lymphoma kinase (ALK) mutation, is able to efficiently block uveal metastasis (15-17). In regards to gefitinib, there is data that indicate that it can be used efficiently for blocking choroidal metastasis as well as for treating choroidal metastasis (18). Moreover, it has been previously reported that several molecular pathways are deregulated, usually by overexpression, which induces TKI resistance. The same pathways have been identified in uveal metastasis of a lung adenocarcinoma under conditions of treatment with TKI (19, 20). It has been observed that afatinib, which is a second generation TKI, has been used to effectively treat and control brain metastasis (21). In our institute we recently diagnosed a patient with adenocarcinoma (a 50-year-old woman, non-smoker, and EGFR positive) and we administered afatinib. Upon diagnosis, the patient was identified to be at stage IV due to 3 metastatic sites in the brain with local edema (an oral suspension of dexamethasone was also initiated). She received afatinib (40 mg) and brain irradiation. Due to severe adverse effects, which were mostly gastrointestinal, the dosage was lowered to 20 mg within 60 days of initiation (Figure 1).

However, eye puffiness slowly developed around the right eye and an MRI revealed local tissue. The repeat biopsy also revealed an adenocarcinoma without EGFR mutation. Although we could have changed the treatment to chemotherapy, we considered this progress an oligometastatic disease progression and decided to continue with afatinib until disease progression in the primary site. We decided to evaluate the primary site within the following two months (two months after diagnosis and treatment initiation with the TKI). Based on the current data re-biopsy of the eye metastasis should be performed if there is an easy approach. The tissue could provide us information regarding the biological behavior of the metastatic site and future solutions for treatment. For example in our case if T790 is observed then osimertinib could be considered. The major issue that still remains is whether we should biopsy sites other than the primary cancer site or biopsy metastatic sites as a gold standard for the approach to treatment (16, 22-30).
